# Construction and application of a time-saving mode in China for the treatment of acute ischemic stroke

**DOI:** 10.3389/fneur.2024.1367801

**Published:** 2024-03-19

**Authors:** Yazhou Yan, Li Du, Xiu Shangguan, Lujun Li, Yuxiang Chi, Yu Wang, Shuai Cheng, Qinghai Huang, Yuan Pan, Tao Xin

**Affiliations:** ^1^Stroke Center, No. 971 Naval Hospital of PLA, Qingdao, China; ^2^Department of Neurovascular Center, Changhai Hospital Affiliated to the Naval Medical University, Shanghai, China

**Keywords:** acute ischemic stroke, intravenous thrombolysis, green channel, emergency treatment, effectiveness

## Abstract

**Objective:**

To explore the construction and application in the practice of green channel in No. 971 Naval Hospital of PLA (No. 971 Hospital mode) for the treatment of acute ischemic stroke (AIS).

**Methods:**

This retrospective study involved a cohort of 694 suspected stroke patients from December 2022 to November 2023 undergoing emergency treatment for stroke at our institution. Among them, 483 patients were treated with standard green channel (the control group), and 211 patients adopted the No. 971 Hospital mode for treatment (the study group). The biggest difference between the two groups was that the treatment process started before admission. We compared the effectiveness of the emergency treatment between the two groups and the thrombolysis treatment.

**Results:**

Compared with control group, the accuracy rate of determining stroke and the rate of thrombolysis were significantly higher (*p* = 0.002, 0.039) and the door to doctor arrival time (DAT) and the door to CT scan time (DCT) of the study group was significantly shorter (all *p* < 0.001). There were 49 patients (10.1%) and 33 patients (15.6%) from the control group and study group receiving thrombolysis, respectively. The DAT, DCT, imaging to needle time (INT), and door to needle time (DNT) of patients receiving thrombolysis in the study group were significantly shorter than that in the control group (all *p* < 0.01). The NIHSS in the study group after the thrombolysis was lower than that in the control group (*p* = 0.042).

**Conclusion:**

No. 971 Hospital model can effectively shorten DAT, DCT, INT, and DNT, and improve the effectiveness of thrombolysis and prognoses of AIS patients.

## Background

Stroke has become a main cause of death and disability in China (1), and ischemic stroke accounts for more than 70% of all stroke (2). Intravenous thrombolysis and endovascular treatment (EVT) have become the standard therapies for acute ischemic stroke (AIS) with large-vessel occlusion, and the effect of these treatments is significantly correlated with time (3, 4). It has been reported that each minute saved in onset-to-treatment time for AIS with large-vessel occlusion granted on average 4.2 days of extra healthy life (5). However, in China, only a small number of AIS patients could reach the emergency department within 3 h after onset. Therefore, shortening the reperfusion time and enabling more people to undergo thrombolysis and EVT within the time window are of great significance. The stroke center of our institution has utilized information technology to construct the green life-safety channel (No. 971 Hospital mode), moving part of the hospital’s treatment process forward to the ambulance to further shorten the thrombolysis time. Hence, we conducted this retrospective study to analyze the impact of “No. 971 Hospital mode” on the treatment and prognosis of AIS patients.

## Materials and methods

### Patients

We retrospectively reviewed all 694 suspected stroke patients who received green channel treatment for stroke at our institution from December 2022 to November 2023. 483 patients treated with standard green channel from December 2022 to June 2023 were divided into control group, and 211 patients from July 2023 to November 2023 were enrolled into the study group, who adopted the No. 971 Hospital mode for treatment. The inclusion criteria were: (1) age ≥ 18 years; (2) during the pre-examination, the highly suspected stroke including one of the following symptoms suddenly appeared: weakness or numbness of one side of body, one side of facial numbness or distortion of commissure, barylalia or language understanding difficulty, gaze with both eyes, loss or blurring of vision, dizziness with vomiting, previously rare severe headache, consciousness disorder or convulsion; (3) within 6 h after onset; (4) according to Chinese Guidelines for Diagnosis and Treatment of Acute Ischemic Stroke (2018), patients treated with intravenous thrombolysis were with National Institutes of Health Stroke Scale (NIHSS) score>5 and ≤ 25 (6). The exclusion criteria were: (1) patients who had contraindications to intravenous thrombolysis; (2) another stroke etiology such as dissection, moyamoya disease, or vasculitis; (3) patients without complete medical records. Patients’ demographics (age and gender), risk factors (hypertension, diabetes, hyperlipemia, smoking, and drinking), clinical characteristics, and treatment results were collected. This retrospective study was approved by our hospital institutional review board and the need for informed consent was waived.

### Treatment details

**Figure 1:**
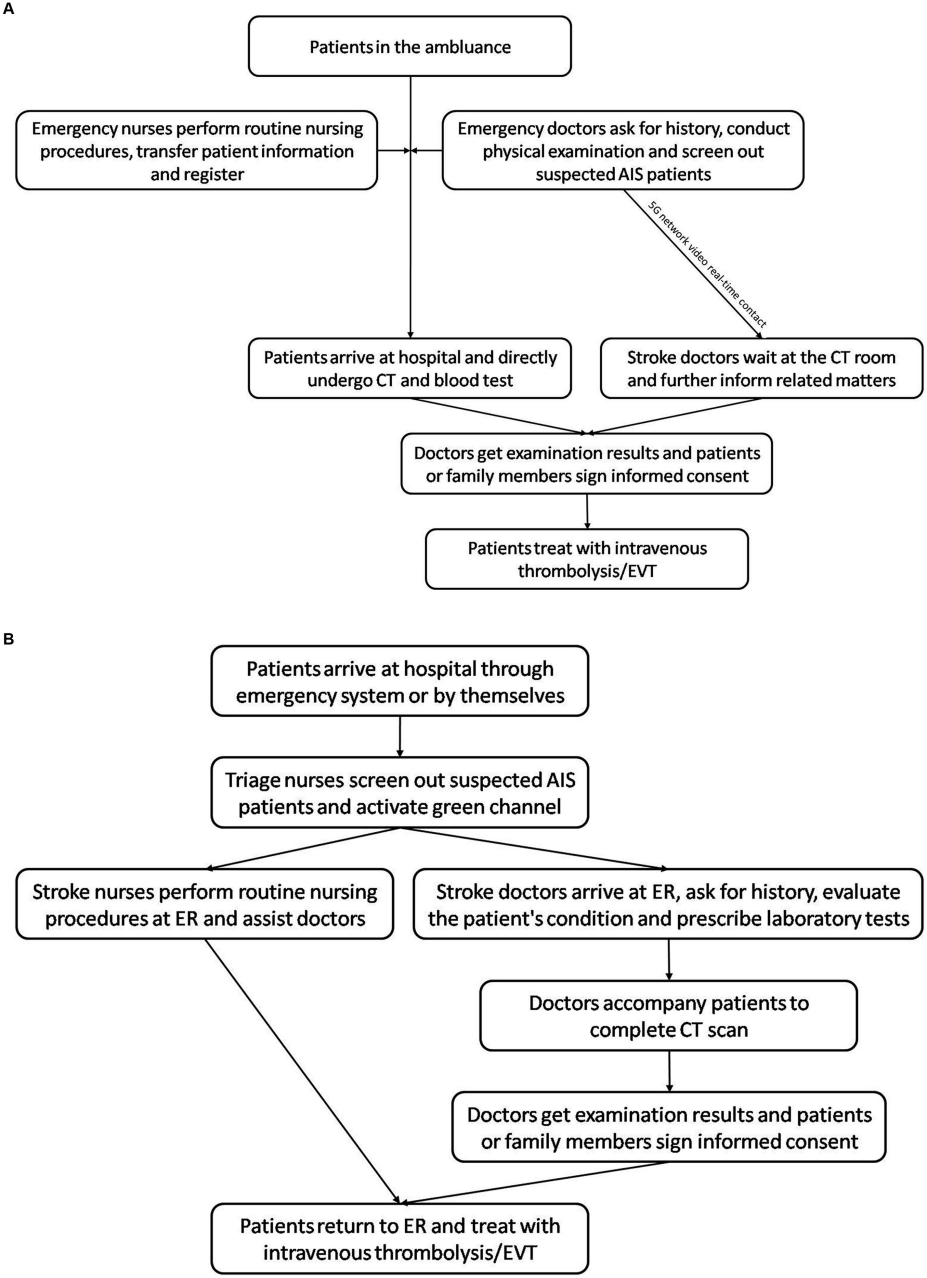
Flow chart showing the emergency treatment modes. **(A)** Green channel in No. 971 Naval Hospital of PLA. **(B)** Standard green channel in China. AIS, acute ischemic stroke; CT, computed tomography; EVT, endovascular treatment; ER, emergency room.

In the study group, the biggest difference from the standard green channel was that the treatment process of No. 971 Hospital mode started before admission. In the ambulance, the emergency doctors collected the medical history, conducted the physical examination, screened out suspected acute stroke cases and made the video contact with the hospital stroke doctors through the 5G network to preliminarily judge the condition. The nurses performed routine procedures, including oxygen inhalation, blood pressure measurement, blood glucose measurement, establishing venous access, drawing blood and electrocardiogram measurement, and connect to the hospital to complete the patient registration and information transmission work. At the same time, the doctors informed the patients or family members of the related matters and potential risks of thrombolysis and EVT. After entering the hospital, the patient was directly transferred to the examination department to complete CT examination and blood test, and so on. After CT scan, the stroke doctors immediately decided whether to carry out intravenous thrombolytic therapy and required the informed consent. Meanwhile, for patients with high NIHSS score (>8 points), doctors immediately performed digital subtraction angiography (DSA) to evaluate the intracranial vessels and prepared for EVT. All medical procedures were post-paid (Figure 1A).

In the control group, the patients underwent the treatment of standard green channel. Patients came to the hospital through the emergency system or by themselves. In the emergency department, stroke nurses conducted pre-examination, triage and registration, and activated the green channel. Then, they informed the stroke doctors and established venous access. The stroke doctors went to the emergency room to evaluate the patient’s condition, prescribe laboratory tests, and perform the imaging examinations. Then, the doctor accompanied the patient to complete the CT scan, conducted a conversation before the thrombolysis and performed intravenous thrombolysis in the emergency department after signing the informed consent (Figure 1B).

### Clinical assessment and follow-up

Evaluation of the effectiveness of the emergency treatment: (1) the onset to door time (ODT, the time from the patients realizing they were sick to arrival at hospital), (2) the door to doctor arrival time (DAT, the time from patient arrival at hospital to stroke doctor reception), (3) the door to CT scan time (DCT, the time from patient arrival at hospital to the beginning of CT scan), (4) the imaging to needle time (INT, the time between completion of CT scan and patient receiving thrombolysis), (5) the door to needle time (DNT, the time between patient arrival at hospital and receiving thrombolysis). The accuracy rate of determining stroke was also used as an evaluation index. Accuracy rate of determining stroke = (number of suspected stroke-number of non-stroke) / number of suspected stroke×100%. The neurological deficit scores (NIHSS) were used to assess the neurological deficit levels before and after the thrombolytic therapy (7). Clinical outcome was assessed also using NIHSS score at 3-month follow-up at the clinic or by phone interview, and then 6 months thereafter.

### Statistical analysis

For normally distributed continuous variables (described as mean ± SD), analysis was performed using Student’s *t-*test, while for nonnormally distributed continuous variables (described as median and quartile), analysis was performed using the Mann–Whitney *U* test. Categorical variables were reported as proportions and analyzed by the chi-square test. A *p*-value ≤0.05 was considered statistically significant. Statistical analysis was performed using standard software (SPSS Version 21.0; IBM, Armonk, NY, USA).

## Results

### Comparison of the effectiveness of emergency procedures between the two groups

In the control group, a total 483 suspected patients were included, including 82 patients (17.0%) who were diagnosed as non-stroke by stroke doctor, 49 patients (10.1%) receiving thrombolysis, 96 patients (19.9%) with intracranial hemorrhage, 63 patients (13.0%) who refused examination or treatment, 14 patients (2.9%) who underwent EVT without thrombolysis, 151 patients (31.3%) beyond the time window, and 28 patients (5.8%) with transient ischemic attack (TIA); while there were 211 suspected patients in the study group, including 17 patients (8.1%) diagnosed as non-stroke by stroke doctor, 33 patients (15.6%) receiving thrombolysis, 44 patients (20.9%) with intracranial hemorrhage, 23 patients (10.9%) refusing examination or treatment, 10 patients (4.7%) treated EVT without thrombolysis, 74 patients (35.1%) beyond the time window, and 10 patients (4.7%) with TIA.

In the study group, the rate of thrombolysis and the accuracy rate of determining stroke were significantly higher than that in the control group (*p* = 0.039, 0.002). Compared with control group, the DAT of the study group was significantly shorter (1.2 ± 0.9 min vs. 7.8 ± 3.1 min, *p* < 0.001). Eventually, 360 patients (74.5%) and 176 patients (83.4%) from the control group and study group underwent CT scans, respectively. And, the DCT of study group was 5.7 ± 2.5 min (range, 1 to 13 min), which was significantly shorter than that (24.8 ± 7.9 min; range, 5 to 54 min) in control group (*p* < 0.001). There was no significant difference in ODT between the two groups (*p* = 0.052). Above data has been summarized in Table 1.

**Table 1:** Effectiveness of emergency procedures between two groups.

Variable	Control group (*n* = 483)*	Study group (*n* = 211)*	*p-*value
Etiology			
Thrombolysis	49 (10.1)	33 (15.6)	**0.039**
Non-stroke	82 (17.0)	17 (8.1)	**0.002**
Intracranial hemorrhage	96 (19.9)	44 (20.9)	0.768
Refuse examination or treatment	63 (13.0)	23 (10.9)	0.431
EVT without thrombolysis	14 (2.9)	10 (4.7)	0.222
Beyond time window	151 (31.3)	74 (35.1)	0.324
TIA	28 (5.8)	10 (4.7)	0.573
Accuracy rate of determining stroke	401 (83.0)	194 (91.9)	**0.002**
Various treatment times			
ODT (min)	147 ± 25.7	143 ± 24.5	0.052
DAT (min)	7.8 ± 3.1	1.2 ± 0.9	**<0.001**
DCT (min)	24.8 ± 7.9	5.7 ± 2.5	**<0.001**

*Values are mean ± SD or number of patients (percentage). The bold entries mean statistically significant (*p* < 0.05). EVT, endovascular treatment; TIA, transient ischemic attack; ODT, onset to door time; DAT, doctor arrival time; DCT, door to CT scan time.

### Comparison of the baseline characteristics and effectiveness of thrombolysis treatment between the two groups

There were 49 patients and 33 patients from the control group and study group receiving thrombolysis alone, although some patients underwent angiography evaluation. There were no significant differences in the baseline data and ODT of the patients receiving thrombolysis between the two groups (all *p*>0.05, Table 2).

**Table 2:** Clinical data and the effectiveness of thrombolysis treatment between two groups.

Variable	Thrombolysis in control group (*n* = 49)*	Thrombolysis in study group (*n* = 33)*	*p-*value
Age (years)	59.1 ± 11.6	63.5 ± 10.2	0.080
Male	30 (61.2)	19 (57.6)	0.741
Hypertension	24 (49.0)	18 (54.5)	0.621
Diabetes mellitus	8 (16.3)	3 (9.1)	0.346
Hyperlipemia	9 (18.4)	4 (12.1)	0.448
Smoking	10 (20.4)	11 (33.3)	0.189
Drinking	7 (14.3)	2 (6.1)	0.243
Various treatment times			
ODT (min)	143 ± 24.7	138 ± 25.1	0.364
DAT (min)	7.5 ± 2.6	1.1 ± 1.0	**<0.001**
DCT (min)	24.6 ± 7.0	5.6 ± 2.4	**<0.001**
INT (min)	18.3 ± 9.0	12.9 ± 6.7	**0.004**
DNT (min)	47.8 ± 13.8	25.7 ± 8.0	**<0.001**
NIHSS at admission	10 (8, 12)	12 (8, 14)	0.076^#^
NIHSS after thrombolysis	6 (5, 7)	5 (4, 7)	**0.042** ^#^
NIHSS at follow-up	3 (2, 4.5)	3 (1, 5)	0.194^#^

*Values are mean ± SD, number of patients (percentage), or mean (P_25_, P_75_). The bold entries mean statistically significant (*p* < 0.05). ^#^Mann–Whitney *U* test. ODT, onset to door time; DAT, doctor arrival time; DCT, door to CT scan time; INT, imaging to needle time; DNT, door to needle time; NIHSS, National Institutes of Health Stroke Scale.

As shown in Table 2, the DAT, DCT, INT, and DNT in the study group were significantly shorter when compared with the corresponding times in the control group (all *p* < 0.01). The NIHSS in both groups after the thrombolysis were declined when compared with that at admission (both *p* < 0.05) and the NIHSS in the study group after the thrombolysis was lower than that in the control group (*p* = 0.042).

The mean clinical follow-up time of these patients of control group and study group were 10.9 ± 2.1 months (range 7 to 15 months) and 5.1 ± 1.2 months (range 7 to 15 months), respectively. There was no significant difference of NIHSS score at the latest clinical follow-up between the two groups (*p* = 0.194).

## Discussion

AIS has the characteristics of rapid onset and quick progression, with high risk of morbidity and mortality. The treatment guidelines for the AIS at home and abroad have put forward the concept of “time is the brain,” which means that emergency clinician must carry out the treatment process of AIS as soon as possible, and quickly complete the patient identification, transfer, triage, condition evaluation, treatment plan formulation, treatment, and transfer to the stroke monitoring unit (8). At present, the green life-safety channel for emergency care has provided great convenience for all patients in critical and serious conditions, giving them priority in all aspects of treatment and providing valuable time for rescue. But in fact, even if the AIS patients have the priority, the emergency treatment process is not decreased, and the patients’ families hesitate to make the treatment decision in a short time, which may delay treatment. The No. 971 Hospital mode proposed in this study further compresses the diagnosis and treatment time on the way of out-of-hospital transfer of patients and makes the patient engagement process lead by the clinician, which shorten the intermediate stage time and ensure the patients receive prehospital treatment. Also, due to the doctors in the ambulance contacting stroke doctors through the network to make the diagnosis together, the accuracy rate of determining stroke in the study group was significantly higher than that in the control group, which could save medical resources.

Intravenous thrombolysis is the essential treatment for AIS and can significantly decrease the rates of disability and mortality and improve prognoses (9). But, there is a strict requirement on the thrombolytic therapy time window. Therefore, the timeliness of treatment and the complete AIS rescue system in the hospital are the key to make patients undergo thrombolysis and EVT as soon as possible. The DNT is the most critical index in evaluating the treatment effectiveness of hospital intravenous thrombolysis for AIS patients, and according to the American Heart Association/American Stroke Association, DNT should be controlled within 60 min as much as possible (10). Threlkeld et al. (11) reported that DNT could be significantly and substantially shortened by the multidisciplinary and collaborative interventions of thrombolysis process, and did not increase the risk of adverse events. In this study, for the No. 971 Hospital mode the medical staff in the ambulance contacted the stroke doctor before admission, so that the stroke doctor could wait early at the CT room, and the patients was directly transferred to the CT room without undergoing emergency department. Also, the thrombolysis room with alteplase was established next to the CT room. All these factors led to the significantly shorter DAT, DCT, and DNT in the study group compared to the control group. The DNT in the study group has reached 25.7 ± 8.0 min.

With shorter DNT in the study group, the rate of thrombolysis was higher than that in the control group, which allowed more AIS patients to receive intravenous thrombolysis. Previous studies have also shown that during pre-hospital and in-hospital emergency medical treatment, implementing targeted interventions related to thrombolysis, including direct CT transfer and administering alteplase in CT, can help reduce delays in the treatment of AIS patients, and increase the proportion of patients receiving intravenous thrombolysis (12, 13). In this study, the emergency doctors informed the patients and family members of the related matters about the thrombolysis and EVT in the ambulance to make them understand the severity of the disease and the importance of thrombolysis and EVT, giving them more time to consider and make decisions. Therefore, the INT and DNT of the study group were significantly shorter than that of the control group. The results of this study showed that No. 971 Hospital mode can minimize the delay in thrombolysis caused by patient’ family decision-making, signing informed consent, etc. during the treatment process, which may shorten the reperfusion time, protect the function of ischemic and hypoxic brain tissue, and prevent ischemia–reperfusion injury. And in this study, the NIHSS score in the study group after thrombolytic therapy was significantly lower than that in the control group. However, different from NIHSS score after thrombosis, there was no significant difference of NIHSS score at the latest clinical follow-up between the two groups. It may be that the patients in the control group had a longer clinical follow-up time, and the duration of rehabilitation exercise was longer than that of the study group. Therefore, the patients of control group may recover better. In addition, the patients with high NIHSS score was performed DSA evaluation as much as possible and the extension of the time window for EVT have provided more patients with opportunities for EVT (4, 14). Therefore, in the treatment process, the patient did not delay the treatment effectiveness because of the suspected cerebral hemorrhage or excessive thrombolysis time window.

There were several limitations of our study, including its retrospective design and the limited sample size from a single center. And the clinical and angiographic follow-up outcomes were needed further analysis. Furthermore, the CT room in our hospital is located at a certain distance from the emergency department, which is also a factor that can significantly reduce DCT time with the No.971 Hospital mode.

## Conclusion

In summary, the emergency “No. 971 Hospital mode” in the treatment of AIS patients can effectively shorten the time from admission to receiving intravenous thrombolysis treatment and improve the effectiveness of thrombolysis, thus improving patients’ neurological functions to a greater extent. It is worthy of clinical application.

## Data availability statement

The original contributions presented in the study are included in the article/supplementary material, further inquiries can be directed to the corresponding authors.

## Ethics statement

The studies involving humans were approved by the Ethics Committee of the No. 971 Naval Hospital of the People’s Liberation Army. The studies were conducted in accordance with the local legislation and institutional requirements. Written informed consent for participation was not required from the participants or the participants’ legal guardians/next of kin in accordance with the national legislation and institutional requirements.

## Author contributions

YY: Conceptualization, Data curation, Formal analysis, Investigation, Methodology, Resources, Software, Validation, Writing – original draft. LD: Data curation, Investigation, Methodology, Resources, Writing – original draft. XS: Data curation, Methodology, Resources, Software, Writing – original draft. LL: Methodology, Resources, Software, Writing – original draft. YC: Data curation, Formal analysis, Methodology, Writing – original draft. YW: Data curation, Investigation, Methodology, Writing – original draft. SC: Methodology, Software, Visualization, Writing – original draft. QH: Funding acquisition, Supervision, Validation, Writing – review & editing. YP: Conceptualization, Supervision, Validation, Visualization, Writing – review & editing. TX: Conceptualization, Funding acquisition, Supervision, Validation, Writing – review & editing.
